# Is Mandarin Chinese a Truth-Based Language? Rejecting Responses to Negative Assertions and Questions

**DOI:** 10.3389/fpsyg.2016.01967

**Published:** 2016-12-20

**Authors:** Feifei Li, Santiago González-Fuente, Pilar Prieto, M.Teresa Espinal

**Affiliations:** ^1^Department of Catalan Philology, Universitat Autònoma de Barcelona (UAB)Barcelona, Spain; ^2^Prosodic Studies Group, Department of Translation and Language Sciences, Universitat Pompeu Fabra (UPF)Barcelona, Spain; ^3^Institució Catalana de Recerca i Estudis Avançats (ICREA)Barcelona, Spain

**Keywords:** truth-based language, REJECT, disagreement, negative propositions, Mandarin Chinese

## Abstract

This paper addresses the central question of whether Mandarin Chinese (MC) is a canonical truth-based language, a language that is expected to express the speaker's disagreement to a negative proposition by means of a negative particle followed by a positive sentence. Eight native speakers of MC participated in an oral Discourse Completion Task that elicited rejecting responses to negative assertions/questions and broad focus statements (control condition). Results show that MC speakers convey reject by relying on a combination of lexico-syntactic strategies (e.g., negative particles such as *bù, méi(yǒu)*, and positive sentences) together with prosodic (e.g., mean pitch) and gestural strategies (mainly, the use of head nods). Importantly, the use of a negative particle, which was the expected outcome in truth-based languages, only appeared in 52% of the rejecting answers. This system puts into question the macroparametric division between truth-based and polarity-based languages and calls for a more general view of the instantiation of a reject speech act that integrates lexical and syntactic strategies with prosodic and gestural strategies.

## Introduction

The parametric division between truth-based and polarity-based languages refers to a classification of answering systems to negative polar questions. A *polar question* is one to which the expected answer is the equivalent to *yes* and *no* (therefore, they are also referred to as *yes-no* questions) (Dryer, 2013). Polar questions can be either positive or negative. *Negative questions* are non-neutral or biased questions (Reese, 2006): “the questioner is biased either toward a positive sentence answer based on the original belief or a negative sentence answer based on the subsequent doubt” (Jones, 1999:8). If the question has a bias the responder can either accept or reject the bias of the question. Thus, if the question has a negative bias, a negative sentence answer accepts the bias, but a positive sentence answer counters it. Negative questions are used with a negative bias and thus convey a negative sentence as the most relevant answer.

Now, at the time of replying to a biased negative question, some languages –so-called *polarity-based languages* (like English)–use an answer to a negative question in the same way as to a positive one; that is, in accordance with the polarity of the sentence answer: if the sentence answer is negative the particle is expected to be negative, and if the sentence answer is positive the particle is expected to be positive. This is exemplified in the English sequences in (1) [Jones, 1999:9, ex. (16)].

(1) Q. Aren't you staying?
A_1_.No [, I'm not].
A_2_.Yes [, I am].

Other languages, however, so-called *truth-based languages* (like Japanese, Korean, Afrikaans, etc.; Holmberg, 2016), use different expressions for replying to negative and to positive questions. Thus, when speakers respond to negative biased questions and wish to express agreement with the implied negative proposition of the question, (s)he uses a positive particle in combination with a negative sentence answer (i.e., I agree = it is true, not *p*). When (s)he wishes to express disagreement with the implied negative sentence (s)he chooses a negative particle in combination with a positive sentence (i.e., I disagree = it is false, *p*). This agree / disagree system (Kuno, 1973; Pope, 1976; Sadock and Zwicky, 1985) is illustrated in the Japanese example in (2) [Jones, 1999: 10, ex. (19)].

(2)     Q. Kyoo wa    atuku nai         desu  ne?
              today part   hot     be+neg  pol    aff
                ‘It isn't hot today, is it?
           A_1_. Hai soo                                    desu  ne.
                  yes pro [ = kyoo wa atuku nai]  pol   aff
                  ‘No, it isn't hot’.
            A_2_. Iie, kyoo wa    atui  desu.
                   no today part  hot   pol
                   ‘Yes, it is hot today.’

Pope (1976) already warns the reader that no language will have a completely straightforward system, and in recent independent studies (González-Fuente et al., 2015; Roelofsen and Farkas, 2015; Claus et al., 2016; Holmberg, 2016) it has been argued that this typology corresponds to idealized models and overall they call into question the parametric distinction between truth-based vs. polarity-based systems. Roelofsen and Farkas (2015) argue that English and other languages such as Romanian, Hungarian, French and German are not completely polarity-based. English “*yes* may be used to signal that the response is positive, or that it agrees with the antecedent possibility in terms of content and polarity, while *no* may be used to signal that the response is negative, or that it reverses the antecedent possibility in terms of content and polarity (Roelofsen and Farkas, 2015:383). Languages such as Hungarian, Romanian and German have been described as having ternary polarity particle systems (Farkas, 2009, 2010, 2011; Farkas and Bruce, 2011; Krifka, 2013; Claus et al., 2016). González-Fuente et al. (2015) show that speakers of Catalan, a language that has been described as being polarity-based, can make use of lexico-syntactic strategies characteristic of truth-based systems at the time of expressing reject. Moreover, Russian (a language with a mixed system that uses polarity-based, truth-based, and echoic strategies) is shown to share with Catalan gestural strategies in the expression of reject (strong repeated head nod and tilt). Overall, one important conclusion from these studies is that the classification between polarity-based and truth-based languages has to be further refined.

In order to do that, in this study we focused on Mandarin Chinese (MC), a Sino-Tibetan language that has been claimed (Jones, 1999; Holmberg, 2016) to function on a truth-value basis in responding to negative polar questions.^1^ We seek to experimentally investigate the following questions: (i) Is MC a truth-based language? (ii) Does MC support a universal answering system based on the instantiation of two cognitive operators: a reject operator and an assert operator (in line with Krifka, in press)? And (iii) how does MC instantiate these operators and what is the role of prosody and gesture in the just mentioned universal answering system (González-Fuente et al., 2015)? We hereby aim at exploring whether MC shares with the just mentioned natural languages, namely Catalan and Russian, some strategies expressing reject.

This article is organized as follows. Section Methods presents the methods and materials of our experiment. Section Results presents the results of our production experiment. Section Discussion presents the theoretical framework on which we base our analysis and discusses our findings in relation to the typological distinction between polarity-based and truth-based languages and also in relation to the hypothesis that languages resort to various complementary strategies at the time of expressing reject.

## Methods

A production experiment with 8 native speakers of MC was conducted within the premises of the Universitat Pompeu Fabra. These speakers participated in a Discourse Completion Task (henceforth DCT) that aimed to elicit semi-spontaneous and contextualized rejecting responses to negative assertions/questions and broad focus statements.

### Participants

Eight native speakers of MC (7 women and 1 man; mean age = 25.75) participated in the DCT. All of them were from China, but recruited in Barcelona.^2^ According to their replies to a sociolinguistic test they were exposed to, they have been living in Barcelona between seven months and 4 years (mean = 1 year and 4 months), and they speak MC with their friends, colleagues and families in their everyday lives (mean of 3 h/day).

### Materials

The DCT production task was aimed at obtaining semi-spontaneous (and pragmatically controlled) rejecting responses to negative assertions and biased negative questions (critical condition) and broad focus statements (control condition) in MC. In order to achieve that aim, we devised a DCT containing a set of 3 different discourse contexts (library context, delivery package context and wedding context) in 2 different experimental conditions, namely rejecting responses to negative assertions and questions (here named REJECT condition) and unsolicited assertions that do not respond to an antecedent clause uttered with a broad focus intonation (here labeled BROAD FOCUS condition). While Table 1 illustrates two of the discourse contexts used in the DCT for the REJECT condition, Table 2 illustrates one of the discourse contexts used for the BROAD FOCUS condition.

**Table 1:** **Sample of two of the discourse contexts that served as a prompt for the REJECT condition (i.e., Negative Assertion and Negative Question)**.

***Situation*****: Every night your classmate, your class monitor and you go to the library to study. You always sit down together at the table in front of the window**.		
***Linguistic prompt***	***Type of linguistic prompt***	***Agreement status of the expected answer***
(a) When you arrive at the library, the class monitor is already there. Ten minutes later, your classmate arrives just when the class monitor goes to check out books. As she sees you sitting there alone, she greets you and says:	Negative Assertion	Rejecting answer
Kàilái bānzhǎng hái méi dào.		
seem class monitor still no arrive		
“It seems that the class monitor hasn't arrived yet.” In order to *deny* what she said, since the class monitor is already there, *what would you say?*		
(b) When you arrive at the library, the class monitor is already there. Ten minutes later, your classmate arrives just when the class monitor goes to check out books. As she sees you sitting there alone, she greets you and says:	Negative Question	Rejecting answer
Bānzhǎng hái méi dào, shì ma?		
class monitor still no arrive is qpart		
“The class monitor hasn't arrived yet, has he?” In order to *contradict* what she said, since the class monitor has already arrived, *what would you say?*		

**Table 2:** **Sample of one of the discourse contexts that served as a prompt for the DCT for the control sentence in the BROAD FOCUS condition**.

***Situation*****: Every night your classmate, your class monitor and you go to the library to study. You always sit down together at the table in front of the window**.	
***Linguistic prompt***	***Status of the answer***
When you arrive at the library, the class monitor is already there. Ten minutes later, your classmate arrives just when the class monitor goes to check out books and you take a break outside the library. She meets you and chats with you. In the course of the conversation, you'd like to tell her that the class monitor has arrived. *What would you say?*	Broad Focus Statement

Importantly, the three discourse contexts used in the DCT were regarded as neutral or not biased in Chinese culture. We selected situations that most young people are familiar with in their everyday lives: having something delivered at home, meeting with a classmate at the library, and giving a red envelope (traditional wedding present) to their friends at a wedding. In order to make the imagined power relation horizontal between them and the informants, we chose roommates, classmates and friends as interlocutors in the contexts.

All the materials used in this experiment (the situations and the linguistic prompts) were written in MC. Participants read the target discourse contexts for each item and then listened to an utterance (the target negative assertion or question) produced by the experimenter, a native speaker of MC. These negative assertions and questions were audio recorded using a PMD660 Marantz professional portable digital recorder and a Rode NTG2 condenser microphone in a quiet room at the Universitat Pompeu Fabra, for the purpose of ensuring that all of the participants heard the imaginary interlocutor's question/assertion with the same acoustic properties and prosodic cues.

Each participant received a complete set of 9 linguistic prompts: 3 discourse contexts × 3 items (two types of negative propositions in the reject condition, either negative assertion or negative question + 1 broad focus statement). See the Supplementary Material.

### Procedure

Following the DCT method proposed in Blum-Kulka et al. (1989) and Félix-Brasdefer (2010), participants were provided with a situational prompt to which they had to respond as spontaneously as possible. Each participant was presented with a randomized set of cards containing the 9 stimuli in two blocks. Each participant had a 5-min break between the two blocks.

The 8 participants were instructed to respond to the discourse context prompts as naturally as possible. Recall that they read the target discourse contexts for each item and then listened to an order as produced by the experimenter, a native speaker of MC. After listening to this utterance, they produced their answer following the instructions they were asked to follow.

A total of 72 responses were obtained, of which 48 corresponded to a REJECT condition and 24 to a BROAD FOCUS condition.

The video recordings of the DCT were conducted at the Linguistics Lab of the Department of Translation and Language Sciences at the Universitat Pompeu Fabra in Barcelona. Each participant signed a consent form at the beginning of the experimental session. We recorded the whole experiment using a PMD660 Marantz professional portable digital player, with a resolution of 720 × 576 pixels, and the sound was sampled at 44,100 Hz using 16-bit quantization. Participants were asked to stand against a white background and in front of a Panasonic AG-HMC41 professional digital video camera.

### Measures and analyses

Four prosodic characteristics of the target DCT answers were obtained automatically with Praat (Boersma and Weenink, 2008): Mean Pitch, Pitch Range, Pitch Variability and Mean Intensity. Gestures were annotated following McNeill (1992) and Allwood's et al. (2005) gestures coding proposal and aligned with the orthographic transcriptions of the video files using ELAN software (Lausberg and Slöetjes, 2009). We coded the following gestures: head movements (e.g., head nod, head shake, head tilt, head turn), eyebrow movements (e.g., eyebrow raising, eyebrow furrowing), shoulder movements (e.g., shoulder shrug), mouth movements (e.g., mouth corner-up, mouth corner-down, mouth stretch).

The prosodic and gestural measures obtained from the analysis with Praat and ELAN were submitted to statistical analysis by means of a Generalized Linear Mixed Model (GLMM) using SPSS software (IBM Corporation Released, 2013).

## Results

This section presents the results of the set of grammatical, prosodic and gesture strategies used by MC native speakers at the time of rejecting negative discourse accessible propositions in contrast to expressing broad focus statements.^3^

### Lexico-syntactic strategies

The 48 rejecting responses to negative assertions and questions (3 discourse contexts x 2 linguistic prompts –negative assertion or negative question– x 8 participants) were coded according to their different grammatical strategies. The following types of rejecting responses were identified:^4^

3. a. *Méi(yǒu)* ‘no’ ^5^ + explanation. (e.g., *Méi(yǒu). Tā qù jièshū le*. ‘No. She went to check out books’).
b. *Bú(shì) (a)* ‘no’ + explanation. (e.g., *Bú(shì). Wǒ zhèngyào gěi nǐ jiù lái le*. ‘No, I was about to give it to her just when you came’).
c. *Méi(yǒu) (ei/a)* ‘no’ + positive sentence + explanation. (e.g., *Méi(yǒu), tā yǐjīng lái le. Xiànzài zài jièshū ne*. ‘No, she has already arrived. (She) is checking out books now’).
d. *Méi(yǒu), méi(yǒu)* ‘no, no’ + positive sentence + explanation. (e.g., *Méi(yǒu), méi(yǒu), tā dào le. Jiè shū qù le*. ‘No, no, she has arrived. (She) went to check out books’).
e. *Bú(shì) (de/a/ei)* ^6^ ‘no’^7^ + positive sentence + explanation. (e.g., *Bú(shì), tā yǐjīng dào le. Qù jièshū le*. ‘No, she has already arrived. (She) went to check out books’).
f. *Nǎ lǐ* ‘no’ + positive sentence + explanation. (e.g., *Nǎ lǐ, wǒ yǐjīng gěi tāmen le. Zhè shì lìngwài de péngyou de*. ‘No, I have already given (it) to them. This belongs to another friend’).
g. Positive sentence + (explanation). (e.g., *Wǒ yǐjīng gěi le. Wǒ shǒushàng de zhè liǎnggè shì q*í*tā rén de*. ‘I have given (it to her). These two in my hand are from other people’).

According to Chinese grammars (Li and Thompson, 1981; Lü, 1999), *bú(shì)* and *méi(yǒu)* can be used as standalone negative answer particles and can also be used as negative adverbs within the sentence. Now, although only one *bú(shì)* and one *méi(yǒu)* is postulated in MC traditional grammar (Lü, 1999),^8^ in our database *bú(shì)* and *méi(yǒu)* have two different functions: as negative answer particles they are situated externally, at the left periphery of the sentence, to which we will refer as *bú(shì)*_1_ and *méi(yǒu)*_1_, whereas as negative adverbs they occur sentence-internally, to which we will refer as *bú(shì)*_2_ and *méi(yǒu)*_2_. Notice that both uses can combine within an utterance, as exemplified in the replies in (4).

(4)     Q: Tā yǐjīng    dào     le,      shì   ma?
               he already arrive  part is     qpart
                ‘He has already arrived, hasn't he?’
          A_1_. Bú(shì),  tā  hái   méi(yǒu)   dào.
                no is        he still no have      arrive
                ‘No, he hasn't arrived yet.’
          A_2_. Méi(yǒu),  tā  hái   méi(yǒu) dào.
                 no have      he still no have    arrive
                ‘No, he hasn't arrived yet.’

Although in the current DCT we did not find combinations of the two uses,^9^ the external and the internal one, we found that 31% of the responses used *méi(yǒu)*_*1*_ as the negative answer particle to reject negative assertions/questions, and 21% of the responses used *bú(shì)*_*1*_ as the negative answer particle to reject negative assertions/questions. The use of *méi(yǒu)*_*2*_ sentence internally corresponds to the negation of the proposition of “he having arrived.”

**Figure 1:**
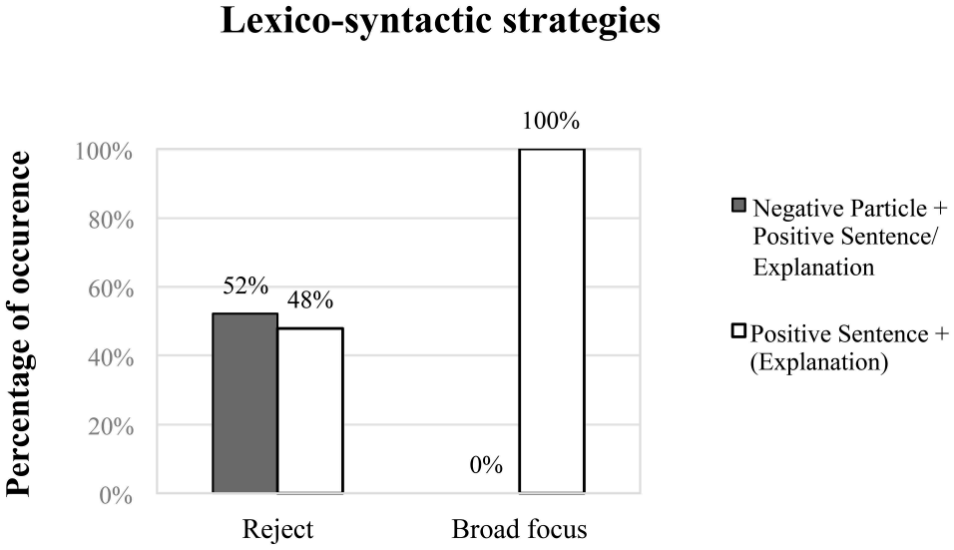
**Percentage of occurrence of lexico-syntactic strategies used by MC speakers in the two conditions (REJECT vs. BROAD FOCUS)**. Negative particles followed by positive sentences/explanation are displayed in gray columns, while positive sentences followed by explanation are displayed in white columns.

The results of the lexico-syntactic strategies in (3) show that for the expression of reject MC speakers rely on two main types of lexico-syntactic strategies: (1) a negative particle followed by a positive sentence or an explanation (in 52% of cases, see examples in (a) through (g) above), which is the expected response for a truth-based language; but also (2) a positive sentence optionally followed by an explanation (in 48% of cases, see the example in (g) above). The latter strategy (a positive sentence optionally followed by an explanation) was the only one employed by MC speakers in broad focus statements (100%). Figure 1 shows the percentage of occurrence of these two strategies used by MC speakers in the two conditions (REJECT vs. BROAD FOCUS).

**Figure 2:**
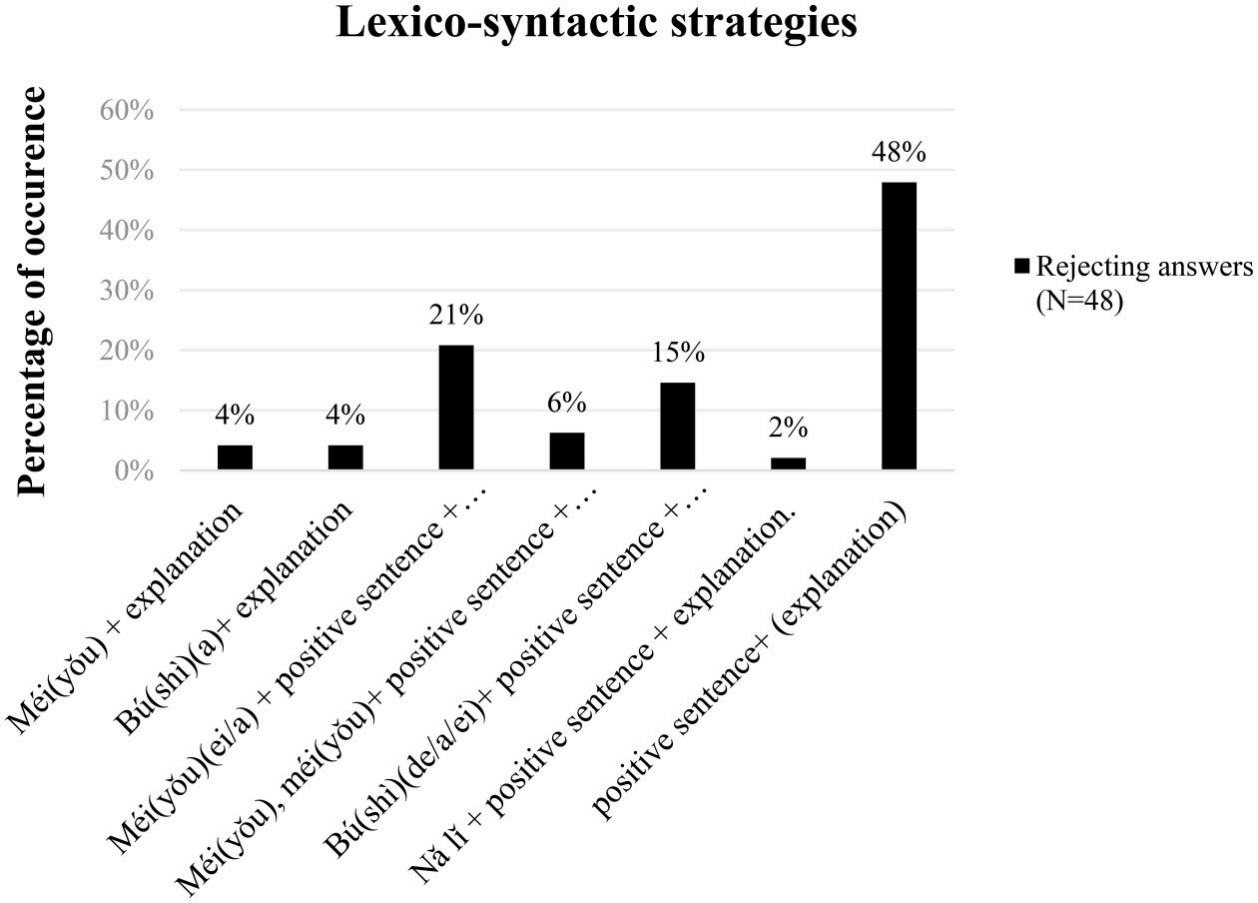
**Percentage of occurrence of the various lexico-syntactic strategies used by MC speakers in the REJECT condition**.

As expected for a truth-based language, rejecting answers to negative assertions and questions were oftentimes produced with negative particles *bú(shì) / méi(yǒu)* “no” answers. Answers with *bú(shì) / méi(yǒu)* “no” (i.e., the particles followed by an explanation or a negative sentence) comprised 52% of rejecting answers to negative assertions and questions, of which 31% of the responses used *méi(yǒu)* as the negative answer particle to reject negative assertions/questions, and 21% of the responses used *bú(shì)* as the negative answer particle to reject negative assertions/questions. The various types of lexical strategies, used for rejecting negative assertions/questions were as follows (listed in order of the frequency in which they were obtained): *Méi(yǒu) (ei/a)* + positive sentence + explanation (21%), *Bú(shì) (de/a/ei)* + positive sentence + explanation (15%), *Méi(yǒu), méi(yǒu)* + positive sentence + explanation (6%), *Méi(yǒu)* + explanation (4%), *Bú(shì) (a)* + explanation (4%), *Nǎ lǐ* + positive sentence + explanation (2%). Importantly, however, 48% of the rejecting answers obtained simply used a positive sentence + (explanation), as illustrated in Figure 2.^10^

It is important to notice that the absence of a particle followed by a positive sentence (and an optional explanation) is not expected among truth-based languages, precisely because it is an echoic strategy (Jones, 1999). However, the absence of lexical means, such as speech act particles, for the expression of reject, might be compensated by the emergence of other non-lexical strategies (i.e., prosodic and gestural strategies). In the next sections we report on the prosodic and gesture strategies used by MC speakers in rejecting responses to negative propositions, in contrast with the ones they used for broad focus statements.

### Prosodic strategies

As results for lexico-syntactic strategies showed two clear strategies for expressing reject, in this section we compare the behavior of several prosodic markers (e.g., Mean Pitch, Pitch Range, Pitch Variability and Mean Intensity) in three different conditions, namely Reject_PS (i.e., Reject responses to negative questions/assertions produced with a positive sentence) vs. Reject_Neg+PS (Reject responses to negative questions/assertions produced with a negative particle followed by a positive sentence) vs. Broad Focus. Four Generalized Linear Mixed Model (GLMM) tests were run with RESPONSE_TYPE as the fixed factor (Reject_PS vs. Reject_Neg_PS vs. Broad Focus), and with Mean Pitch, Pitch Range, Pitch Variability and Mean Intensity set as dependent variables. Subject and utterance were set as random factors.

**Figure 3:**
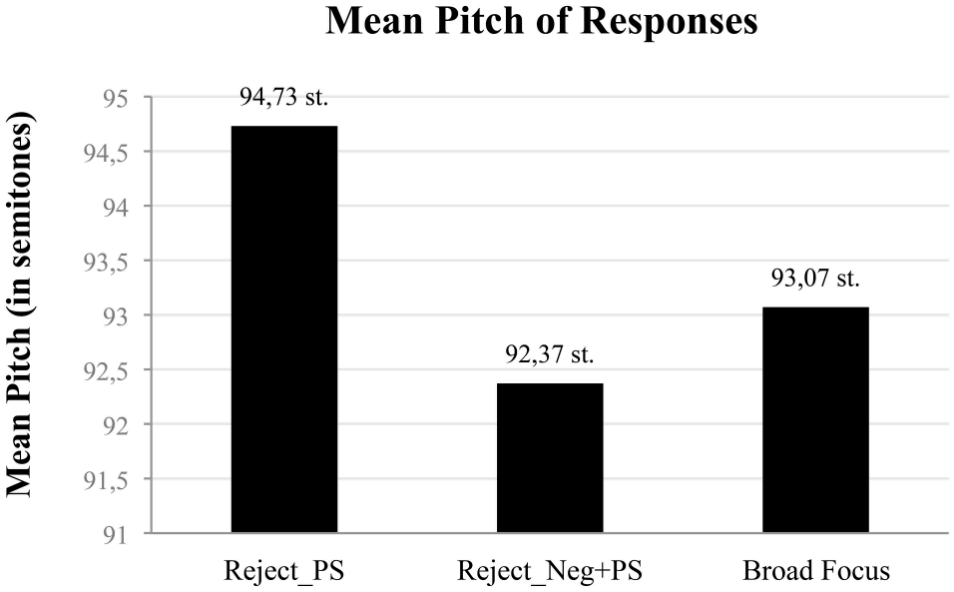
**mean pitch (in semitones) (y-axis) of (1) Reject responses to negative questions/assertions produced with a positive sentence (left column), (2) Reject responses to negative questions/assertions produced with a negative particle followed by a positive sentence (middle column) and (3) Broad Focus responses (right column)**.

Interestingly, results for Mean Pitch showed that MC speakers significantly increase their pitch [*F*_(2, 69)_ = 3.31, *p* < 0.5] when they reject a negative proposition with a positive sentence (see Figure 3, left column) compared to (i) when they reject a negative proposition with a negative particle followed by a positive sentence (Figure 3, middle column), and (ii) when they pronounce a broad focus statement (Figure 3, right column).

In sum, the results in this section show that when MC speakers reject a negative proposition by means of a standalone positive sentence, they produce it with higher mean pitch than when they reject it with a negative particle followed by a positive sentence.

### Gesture strategies

This subsection analyzes the gesture strategies produced with the rejecting responses to negative assertions/questions, contrasting them with the ones produced in broad focus statements. As for the analyses of the prosodic strategies, we compared the gestural characteristics of three different responses' groups: Reject_PS (i.e., Reject responses to negative questions/assertions produced with a positive sentence) vs. Reject_Neg+PS (Reject responses to negative questions/assertions produced with a negative particle followed by a positive sentence) vs. Broad Focus.

A chi-square test confirmed that in general MC speakers produced significantly more gestures (head, eyebrows, shoulders and mouth movements were analyzed) in the reject condition than in the broad focus condition [χ^2^(1) = 27.11, *p* < 0.01]. When MC speakers had to reject a negative proposition they produced one or more gestures in 48% of the responses whereas when they pronounced a broad focus statement they only produced a gesture in 4% of the responses.

**Figure 4:**
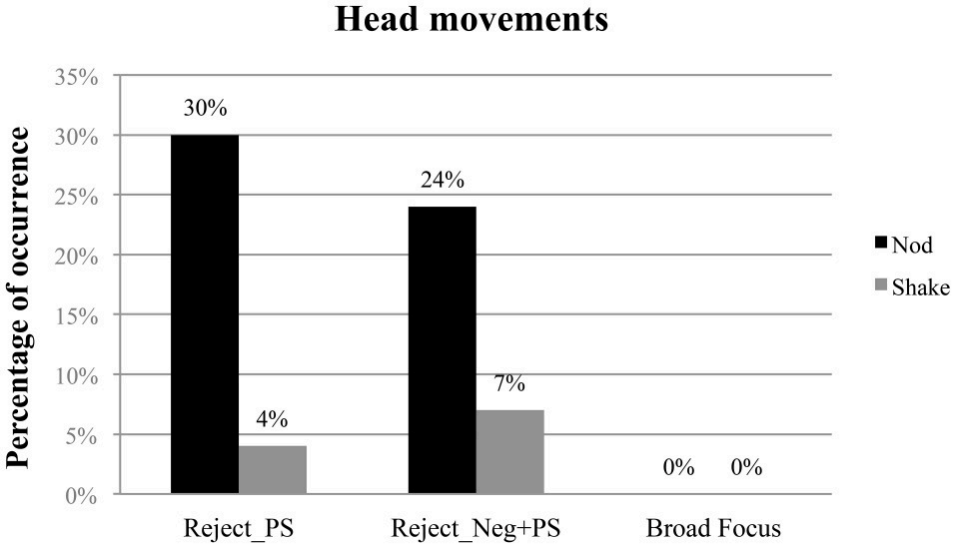
**Percentage of head movements (y-axis) of (1) Reject responses to negative questions/assertions produced with a positive sentence (left columns), (2) Reject responses to negative questions/assertions produced with a negative particle followed by a positive sentence (middle columns) and (3) Broad Focus responses (right columns)**. Results are separated by head movement type, e.g., Head Nods (black columns) and Head Shakes (gray columns).

Head movements occurred in 43% of rejecting responses, eyebrow movements in 23% and mouth movements only in 3% of responses. We now concentrate on the most frequent gestures used by MC speakers in this database, namely head nods and head shakes. Results of two chi-square tests showed a significant difference between both Reject_PS and Reject_Neg+PS responses vs. Broad Focus responses [χ^2^(1) = 18.24, *p* < 0.01 and χ^2^(1) = 10.31, *p* < 0.01, respectively]. Figure 4 shows the percentage of head movements (head nods and head shakes) produced in the three response conditions. The results show the contrast between the two reject responses vs. the broad focus responses in the use of head nods or shakes; importantly not even one nod or shake was found in the broad focus condition. Second, as expected, MC speakers were found to use slightly more head nods when rejecting a negative proposition by means of a positive sentence than when using a negative particle followed by a positive sentence, although the results were not found to be significant.

In general, the results of the analysis of the gesture strategies show that participants produce significantly more gestures (and specifically head nods and head shakes) when they reject a negative assertion or question than when they produce a broad focus statement.

Summing up, the results provided in this section show that MC speakers use a specific set of optional lexico-syntactic strategies to reject negative propositions, as well as an increase in mean pitch and a more frequent use of head nod and head shake movements.

## Discussion

### Modeling speech acts

The analysis of the results in Section Results will rely on a framework for illocutionary acts developed by Cohen and Krifka (2011, 2014), and Krifka (2013, 2015, in press), among other references.

The point of departure is that speech acts are conceived as the key factor for changing commitments by the interlocutors and for triggering changes of commitments, in the sense that a basic speech act is a function from an input commitment to an output commitment. Thus, it is assumed that “in asserting a proposition φ, the speaker takes on a commitment to be responsible for the truth of φ, and in promising to behave in a way described by a proposition φ, the speaker takes on the commitment to behave in that way” (Cohen and Krifka, 2014:48). Similarly, in rejecting a negative assertion the speaker expresses his/her unwillingness to be committed to a certain proposition φ accessible from the discourse, and in rejecting a negative question the speaker denies his commitment to a potential propositional antecedent accessible from the previous question, which suggests that rejecting responses to negative assertions and questions must also be interpreted at the level of speech acts.

The two fundamental notions of this model are *commitment state* (c), modeled as a set of propositions, and *commitment space* (C), modeled as a set of commitment states. Accordingly, a speech act A is, more exactly, a function from an input commitment state to an output commitment state, the outcome being that commitment spaces develop during conversation. That is, in this model commitment spaces are sets of commitment states that are rooted in a (non-empty) commitment state and constrain the admissible continuations of commitment states.

Furthermore, for each commitment state c, there is a common ground CG(c) that consists of a set of propositions that are mutually taken to be true. Hence, commitment states play the role of common ground.

Now, what is the role of linguistic forms (such as lexical particles and prosodic cues) and gestural strategies in such a model? We hypothesize that they encode functions from input commitments to output commitments. This is crucial to understand not only that response particles like *yes* and *no* are analyzed as anaphoric elements that pick up propositional discourse referents introduced by preceding sentences (Krifka, 2013), but also to address the role of prosody and gesture in speech act interaction.

More formally, speech acts are uttered by speakers (S1 and S2). The basic type of speech act is assertion (assert), by means of which speakers are said to express two commitments: one by which S1 first expresses a commitment to a proposition [S1:φ] (that is (s)he takes on responsibility for the truth of a proposition), and a second one by means of which the speaker calls on the addressee (S2) to be also committed to that proposition, with the result that the proposition becomes part of the common ground [φ ∈ CG].

A second type of speech act relevant to our study is a request question. A request speech act with respect to a negative polar question is to be conceived as a meta-speech act in the sense that it applies a request to a speech act of assert a negative proposition and restricts the admissible future moves, the permissible speech acts of confirm and reject.

In the specific case of rejecting responses to negative assertions and questions, which is the topic of our research, we have to consider a third type of speech act, referred to with the label reject. A reject speech act with respect to a previous negative assertion or question is also to be analyzed as a meta-speech act in that it applies over assertions (i.e., the assertion of a negative proposition produced by the speaker S1 in the previous discourse) and restricts the admissible future moves to additional assertions (i.e., the assertion of a positive proposition produced by the speaker S2 in the subsequent discourse), analyzed as commitments of interlocutors for the truth of propositions. We represent this conjunction of speech acts (&) in terms of coordination of ForceP(hrases) where “force” stands for the type of speech act (Krifka, 2015).^11^

Finally, it should be pointed out that (i) propositions are formally represented either as T(ense)P(hrases) (if they correspond to positive sentences) or as Neg(ative)P(hrases) (if they correspond to negative ones); (ii) propositions are turned into speech acts by illocutionary operators (request, reject, assert), which project the syntactic category ForceP.

### The expression of reject in MC

In this section we entertain the hypothesis that the expression of reject in a truth-based language like MC can be analyzed in a similar fashion to the expression of reject in polarity-based and echoic-based languages (González-Fuente et al., 2015). Following Krifka (2013, 2015, in press) and Claus et al. (2016) we assume that: (i) speech acts create spaces of commitments, and by means of them interlocutors may introduce changes of commitments, in a dynamic and dialogical way; (ii) a reject speech act is one by which a speaker opposes to the commitment suggested by the interlocutor, and forces a change of commitment with respect to the common ground; and (iii) a reject speech act applies to an assert speech act, in which the polarity of the sentence is expressed. Furthermore, following González-Fuente et al. (2015), we assume that (iv) prosodic and gesture cues may signal specific relationships between the speaker, the proposition uttered and the common ground, and may convey different epistemic commitments of discourse participants.

According to the results of the DCT, MC speakers reject negative assertions/questions using either a positive sentence optionally followed by an explanation, or a negative particle followed by a positive sentence or an explanation (e.g., *Méi(yǒu)* “no”+ explanation; *Bú(shì) (a)* “no”+ explanation; *Méi(yǒu) (ei/a)* “no”+ positive sentence + explanation; *Méi(yǒu), méi(yǒu)* “no, no” + positive sentence + explanation; *Bú(shì) (de/a/ei)* + positive sentence + explanation; *Nǎ lǐ* “no”+ positive sentence + explanation). Let us consider the negative question in (5) with its meaning representation in (6).

(5)     S1 to S2: Tā hái   méi  lá,i     shì ma?
                           he still no    come is   qpart
                           ‘(S)he hasn't come yet, has (s)he?’

(6)  [_ForceP_ request_S2,S1_ *shì ma* [_ForceP_ assert_S1,S2_ [_NegP_ *tā hái méi lái*]]]

The negative question in (5) presents two potential propositional discourse referents, which means that answers to this question may link to two potential antecedent clauses:

φ = ‘(S)he has come’, corresponding to TP [_TP_ *tā yǐjīng lái le*]
ψ = ¬ ‘(S)he has come’, corresponding to NegP [_NegP_ *tā hái méi lái*]

As presented in Section Results, two of the main rejecting responses found in the DCT have the information structure given in (7) and (8).

(7)     S2 to S1: Méi(yǒu), tā yǐjīng lái          le. ‘Yes, he has.’
                           no have   he already come part
          [_CoordP_  [_ForceP_ reject_S2, S1_ *méi(yǒu)* [_ForceP_  assert_S1, S2_
          [_NegP_ *tā hái méi lái*]]] & [_ForceP_ assert_S2, S1_   [_TP_ *tā yǐjīng*
          lái le]]]
(8)     S2 to S1: Tā yǐjīng       lái       le. ‘Yes, he has.’
                           he already come  part
          [_CoordP_ [_ForceP_ reject_S2, S1_ [_ForceP_ assert_S1, S2_ [_NegP_ *tā hái*
          *méi lái*]]] & [_ForceP_ assert_S2, S1_ [_TP_ *tā yǐjīng lái le*]]]

The speaker S2 can optionally express reject to the negative question in (5) by means of negative particle *méi(yǒu)* that picks up a propositional discourse referent. As illustrated in (7), S2 expresses reject to the negative proposition expressed by the NegP *tā hái méi lái*; that is, the negative particle has a discourse anaphoric relationship with ψ. This reject speech act combines in discourse with an assert speech act, by means of which S2 straightforwardly asserts a commitment to the truth of a positive proposition, namely the TP *tā yǐjīng lái le*. Therefore, S2 utters the conjunction of two speech acts, syntactically corresponding to ForceP. The analysis in (8) is parallel to the analysis in (7) with the only difference that the speech act of reject is not overtly expressed by means of a lexical particle, but rather by non-lexical prosodic and gesture strategies.

On the interpretation side, the conversation moves in (7) and (8) indicate that S2 expects that S1 will incorporate φ, a positive proposition, to the common ground. Therefore, at the output of the conversational move expressed in (5) and the replies in (7) and (8), S1 is expected to assume the truth of φ, as represented in (9).

(9) (…, C) + reject_S2, S1_ [S1: ψ] + assert_S2, S1_ [S2: φ] + [S1 ⊢ φ] + [φ ∈ CG]

A commitment space C, updated by a speech act A of reject the proposition ψ is the set of commitment states in C updated with A, which in its turn is updated by a speech act A' of assert the proposition φ, the effects of which are that S1 is committed to the truth of φ, and φ is incorporated into the CG.

Our results show that in addition to the particles *méi(yǒu)* and *bú(shì)*, some utterances included a repetition of *méi(yǒu), méi(yǒu)*, which also corresponds to the expression of reject. Notice that one of these particles cannot correspond to the expression of assert because there is a positive sentence following the repetition of *méi(yǒu), méi(yǒu)*. This meaning is represented in (10).

(10) [_CoordP_[_ForceP_ reject_S2,S1_ *méi(yŏu) méi(yŏu)* [_ForceP_ assert_S1,S2_ [_NegP_ *tā hái méi lái*]]] & [_ForceP_ assert_S2, S1_ [_TP_ *tā yǐjīng lái le*]]]

However, what is most important is that the expression of reject may be conveyed by other means different from lexico-syntactic strategies, which is relevant from the moment that the truth-based vs. polarity-based typological distinction has been mainly based on lexico-syntactic grounds (Pope, 1976; Jones, 1999). Among the set of complementary strategies, prosodic and gesture mechanisms should be highlighted.

Our results further reveal that MC, being a tone language, uses F0 (pitch) not only for lexical purposes, but also for the indication of discourse relations. Specifically, to distinguish prosodically a positive sentence that is meant to convey reject from a positive sentence that conveys a broad focus statement.^12^

On the other hand, MC speakers use a variety of head movements, mainly head nods and head shakes, in the expression of reject. Interestingly, these specific prosodic and gesture strategies contribute to convey reject in a language where the combination of various strategies, beyond particular lexical items and lexical tone is not expected.

Moreover, a specific comment should be devoted to the use of a head nod together with a positive sentence. Our DCT results show that in MC this gesture in combination with a positive sentence implicates pragmatically reject, and that in the absence of specific lexical particles of rejection it is the expression of this operator.^13^

To sum up, Table 3 offers a schematic summary of the rejecting strategies found in the MC answering system.

**Table 3:** **Speech act analysis of rejecting strategies in MC**.

**REJECTING STRATEGIES**	**reject_S2, S1_**	**assert_S2, S1_**	**φ**
*Méi(yǒu)* + explanation	méi(yǒu) “no”	∅	explanation
Bú(shì) (a) + explanation	bú(shì) (a) “no”	∅	explanation
*Méi(yǒu) (ei/a)* + positive sentence + explanation	méi(yǒu) (ei/a) “no”	∅	positive sentence +explanation
*Méi(yǒu), méi(yǒu)* + positive sentence + explanation.	Méi(yǒu), méi(yǒu) “no, no”	∅	positive sentence +explanation
*Bú(shì) (de/a/ei)* + positive sentence + explanation.	bú(shì) (de/a/ei) “no”	∅	positive sentence +explanation
*Nǎ lǐ* + positive sentence + explanation	nǎ lǐ “no”	∅	positive sentence +explanation
positive sentence + (explanation)	∅	∅	positive sentence+ (explanation)
Any of the above + rejecting prosodic and gesture strategies	prosodic cues (mean pitch) and/or gestures (head nod, head shake)		Any of the above

All in all, the results of this experimental investigation put into question the macroparametric division between truth-based and polarity-based languages, and show that a set of lexical, syntactic, prosodic, and gesture strategies are used to reject negative assertions and questions. In line with González-Fuente et al. (2015) we have shown that different strategies coincide in the expression of rejection, and we interpret this fact as supporting (together with Krifka, in press) the existence of a reject operator that can be instantiated by a set of different strategies across languages.

## Conclusion

Previous research has proposed that languages differ as to how they reject negative propositions and has proposed a contrast between truth-based vs. polarity-based languages. The aim of this paper has been to assess the extent to which and how Mandarin Chinese (a language with a truth-based answering system according to Jones, 1999 and Holmberg, 2016) instantiates rejections to negative propositions by taking into account not only lexico-syntactic strategies, but also prosodic and gestural markers.

A total of 8 speakers of Mandarin Chinese were asked to respond to an oral DCT which contained a set of 72 items in two conditions, namely rejecting responses vs. broad focus sentences. The results showed that Chinese rejecting answers to negative propositions have an optional use of negative particles. Importantly, the results also document a systematic use of a higher pitch and a variety of co-speech gestures in the rejecting condition.

Let us finally go back to the initial questions of this investigation, and let us proceed with the final conclusions:

Q1. Is MC a truth-based language? Our results support the conclusion that Chinese does not constitute a pure truth-based language, but rather is a mixed system, whereby polarity-based and truth-based strategies are used. Similarly, polarity-based languages like Catalan, as well as echoic languages like Russian, have also been found to use a mixed set of strategies (González-Fuente et al., 2015).
Q2. Does MC support a universal answering system? The answer to this question is positive. A reject speech act operator applies over an assert speech act operator (Krifka, in press) and can be realized by means of various lexico-syntactic, prosodic, and gesture strategies. These operators may have a null morphophonological realization. Importantly, this analysis can be successfully applied to polarity-based languages like Catalan or echoic languages like Russian (González-Fuente et al., 2015).
Q3. How does MC realize assert and reject? The answer to this question is by means of various lexico-syntactic (*bù/méi(yǒu)* + positive sentence), prosodic (mean pitch) and gesture (head nod and head shake) strategies.

## Ethics statement

The present research was approaved by the Ethics Committee of the Universitat Autònoma de Barcelona. All experimental subjects signed a written consent form which (a) provided information about the study and (b) obtained the subjects' signature stating his or her agreement to participate in the study, together with an option to withdraw his or her data from the study.

## Author contributions

FL ran the DCT production experiment with native speakers of Mandarine Chinese and is responsible for the descriptive analysis of the materials. SG helped in the design of the experiment, analysis of the materials and is responsible for the quantitative analysis of the results. PP is the leader of the Group of Prosodic Studies and helped in the design of the experiment, the coding scheme, as well as the discussion of the results, together with MTE. MTE is responsible for the research and together with PP is responsible for the design, coding, and discussion of the results.

## Funding

We acknowledge support from the Spanish MINECO (FFI2014-52015-P, FFI2015-66533-P), and from the Generalitat de Catalunya to both the Prosodic Studies Group (2014SGR-925) and the Centre de Lingüística Teòrica (2014SGR-1013). The first author acknowledges a grant from the Chinese Scholarship Council, the second author a MEC-Spain FPU 2012-05893 grant, and the last author an ICREA Academia award.

### Conflict of interest statement

The authors declare that the research was conducted in the absence of any commercial or financial relationships that could be construed as a potential conflict of interest.

## Footnotes

^1^Notice that when responding to positive (neutral) questions the linguistic forms chosen agree with the polarity of the question, which means that the two systems (i.e., polarity-based and truth-based) make identical predictions in this case.

^2^One of these participants comes from the Jiangxi province; another one from the Fujian province; one from the Heilongjiang province; one from Beijing; and four of them come from the Sichuan province. Although these different provinces are linked to different dialect areas (the Jiangxi province is associated with the Gan dialect area; the Fujian province with the Hakka dialect area; Heilongjiang, Beijing and the Sichuan province with the northern dialect area), all of our participants spoke standard Mandarin Chinese in the DCT, as this is the national statutory common language since the twentieth century in China (Huang and Liao, 2007).

^3^Regarding the 24 broad focus statements, the results of the lexico-syntactic strategies show that MC speakers rely on one main type of lexico-syntactic strategy (100%), viz. a positive sentence optionally followed by an explanation.

^4^In MC, there are four tones. The first tone is represented by “—,” the second tone by “/,” the third tone by “∨” and the fourth tone by “\.” *Bù* ‘no’ is pronounced in the fourth tone when it is used alone. It is also pronounced in the fourth tone when it precedes the first tone, the second tone or the third tone. It is pronounced in the second tone when it precedes the fourth tone, e.g., *bú shì*. (Huang and Liao, 2007).

^5^In Mandarin Chinese, *méi* means ‘no’ and *yǒu* means ‘have’. When the two lexical items are used together as the negative answer particle *méi(yǒu)*, the composite word also means ‘no’. In addition, *méi(yǒu)* can be shortened to *méi* without changing its meaning. In spoken Chinese, *méi* is used more often than *méi(yǒu)* (Lü, 1999: 383).

^6^*De/a/ei* are modal particles that can be optionally produced at the end of sentences.

^7^In Mandarin Chinese, *bù* means ‘no’ and *shì* is the copula ‘be’. When the two lexical items are used together as the negative answer particle *bú(shì)*, it can be translated to ‘no’. In our DCT, some speakers produced *bú(shì)* as the negative answer particle when they rejected the negative polar questions. *Bú(shì)* is often the response to questions ending with *shì ma*. *Shì ma* is a question word meaning ‘hasn’t it?' or ‘isn’t it?'. For example, when the form of the negative polar question has *shì ma* at the end: *Bānzhǎng hái méi lái, shì ma?* ‘The class monitor hasn't arrived, hasn't he?’, some speakers produce *bú(shì)* in order to express negation. *Bú(shì)* is used to negate *shì ma* in the question (Li, 2007). It should also be mentioned that Mandarin Chinese speakers often just use *bù* ‘no’ to negate negative propositions, rather than *bú(shì)*. However, in our DCT, they used *bú(shì)*.

^8^In MC grammar only one *bú(shì)* and one *méi(yǒu)* are described (Lü, 1999), which are claimed to have two main functions: they can be used as negative answer particles and also as negative adverbs with scope on the sentence. In our paper, in order to distinguish these two functions, we use *bú(shì)*_1_ and *méi(yǒu)*_*1*_ as negative answer particles, and *bú(shì)*_*2*_ and *méi(yǒu)*_*2*_ as negative adverbs within the sentence.

^9^We found them in an independent DCT, where speakers expressed reject to positive assertions and reject to positive polar questions.

^10^No significant difference was found between the relative proportions of lexico-syntactic strategies used for rejecting negative assertions vs. negative questions, as shown in (i) and (ii):

- Reject a negative assertion:
  - a negative particle followed by a positive sentence or an explanation (25% of cases)
  - a positive sentence optionally followed by an explanation (25% of cases).
- Reject a negative question:
  - a negative particle followed by a positive sentence or an explanation (27% of cases)
  - a positive sentence optionally followed by an explanation (23% of cases).

^11^Recall that, originally, for Rizzi (1997) the term ‘force’ specifies whether the sentence is declarative, interrogative, imperative or exclamative.

^12^The reader should bear in mind that both positive sentences that convey reject and positive sentences that convey a broad focus statement share a final particle *le*, which may have either a modal meaning expressing the change of a situation or a compound meaning (modal meaning plus aspectual meaning) expressing the change of a situation and the completion of an action at the same time. In the MC DCT, 100% of the control sentences that were produced with broad focus intonation contained the final *le* particle. On the basis of the participants' detailed responses, 92% of the rejecting answers to negative assertions/questions are produced with a final *le* particle. The remaining 8% of rejecting answers contained only a rejecting particle.

^13^This result is interesting because it shows a contrast with a polarity-based language such as Catalan and a mixed answering system such as the one exemplified in Russian. See González-Fuente et al. (2015) for the claim that in these languages (slight) head nod is associated with a confirming answer and (strong) head nod with a rejecting one.
