# Supplementary material for: Is Mandarin Chinese a Truth-Based Language? Rejecting Responses to Negative Assertions and Questions
Source: Front Psychol. 2016 Dec 20;7:1967. doi: 10.3389/fpsyg.2016.01967 (PMC5167746; doi:10.3389/fpsyg.2016.01967)
Supplement: Supplementary file 1 [file DataSheet1.pdf]

Supplementary Material

Is Mandarin Chinese a truth-based language? Rejecting responses to negative assertions and questions

Feifei Li, Santiago González-Fuente, Pilar Prieto*, M.Teresa Espinal*

*** Correspondence:** Pilar Prieto: Pilar.Prieto@upf.edu &

M.Teresa Espinal: Teresa.Espinal@uab.cat

APPENDIX

The three discourse contexts

图书馆
Library context

每天晚上，你的同学、班长和你都会去图书馆学习，你们总是一起坐在靠窗的那张桌子。
Every night, your classmate, your class monitor and you go to the library to study. You always sit down together at the table in front of the window.

1. 当你到图书馆时班长已经到了。十分钟后，当班长去借书时，你的同学来了。当她看见你时，跟你打招呼并说：“我猜班长已经到了。”
   你对班长到了的事实予以肯定，你会说什么？
   When you arrive at the library, the class monitor is already there. Ten minutes later, your classmate arrives just when the class monitor goes to check out some books. When she sees you, she greets you and says:

Wǒ	cāi	bānzhǎng	yǐjīng	dào	le.
I	guess	class monitor	already	arrive	PART

   ‘I guess the class monitor has already arrived.’
   In order to confirm that the class monitor has arrived, what would you say?

2. 当你到图书馆时，没有看见你的两个同伴。十分钟后，你的同学来了。当她看见你时，跟你打招呼并说：“我猜班长已经到了。”
   你对班长已来的事实予以否定，你会怎么说？
   When you arrive at the library, you don’t see any of your two colleagues. Ten

minutes later, your classmate arrives. When she sees you, she greets you and says:

Wǒ cāi bānzhǎng yǐjīng dào le.
I guess class monitor already arrive PART
'I guess the class monitor has already arrived.'
In order to deny that the class monitor has arrived, what would you say?

3. 当你到图书馆时，没有看见你的两个同伴。十分钟后，你的同学来了，看你一个人坐在那儿，于是跟你打招呼并说："看来班长还没到。"
你对班长没来的事实予以肯定，你会怎么说？
When you arrive at the library, you don't see any of your two colleagues. Ten minutes later, your classmate arrives. As she sees you sitting there alone, she greets you and says:
Kànlái bānzhǎng hái méi dào.
seem class monitor still no arrive
'It seems that the class monitor hasn't arrived yet.'
In order to confirm that the class monitor hasn't arrived yet, what would you say?

4. 当你到图书馆时，班长已经在那里了。十分钟后，当班长去借书时你的同学来了，她看你一个人坐在那儿，于是跟你打招呼并说："看来班长还没到。"
你对她的话予以反对，因为班长已经到了。你会怎么说？
When you arrive at the library, the class monitor is already there. Ten minutes later, your classmate arrives just when the class monitor goes to check out some books. As she sees you sitting there alone, she greets you and says:
Kànlái bānzhǎng hái méi dào.
seem class monitor still no arrive
'It seems that the class monitor hasn't arrived yet.'
In order to deny what she said, since the class monitor is already there, what would you say?

5. 当你到图书馆时，班长已经在那里了。十分钟后，当班长去借书时你的同学来了，她见到你后，跟你打招呼并说："班长已经到了, 是吗？"
你对班长已到的事实予以肯定，你会怎么说？
When you arrive at the library, the class monitor is already there. Ten minutes later, your classmate arrives just when the class monitor goes to check out some books. When she sees you, she greets you and says:
Bānzhǎng yǐjīng dào le, shì ma?
class monitor already arrive PART is QPART
'The class monitor has already arrived, hasn't he?'
In order to confirm that the class monitor has already arrived, what would you say?

6. 当你到图书馆时，没有看见你的两个同伴。十分钟后，你的同学来了。她见到你时，跟你打招呼并说：“班长已经到了，是吗？”
你对班长已到的事实予以否定，你会怎么说？
When you arrive at the library, you don’t see any of your two colleagues. Ten minutes later, your classmate arrives. When she sees you, she greets you and says:
Bānzhǎng yǐjīng dào le, shì ma?
class monitor already arrive PART is QPART
‘The class monitor has already arrived，hasn’t he?’
In order to deny that the class monitor has already arrived, what would you say?

7. 当你到图书馆时，没有看见你的两个同伴。十分钟后，你的同学来了。看你一个人坐在那儿，于是跟你打招呼并说：“班长还没到，是吗？”
你对班长没到的事实予以肯定，你会怎么说？
When you arrive at the library, you don’t see any of your two colleagues. Ten minutes later, your classmate arrives. As she sees you sitting there alone, she greets you and says:
Bānzhǎng hái méi dào, shì ma?
class monitor still no arrive is QPART
‘The class monitor hasn’t arrived yet, has he?’
In order to confirm that the class monitor hasn’t arrived, what would you say?

8. 当你到图书馆时，班长已经在那里了。十分钟后，当班长去借书时你的同学来了，她看你一个人坐在那儿，跟你打招呼并说：“班长还没到，是吗？”
你对她的话予以反对，因为班长已经到了。你会怎么说？
When you arrive at the library, the class monitor is already there. Ten minutes later, your classmate arrives just when the class monitor goes to check out some books. As she sees you sitting there alone, she greets you and says: “The class monitor hasn’t arrived yet, has he?”
Bānzhǎng hái méi dào, shì ma?
class monitor still no arrive is QPART
‘The class monitor hasn’t arrived yet, has he?’
In order to contradict what she said, since the class monitor has already arrived, what would you say?

9. 当你到图书馆时，班长已经在那里了。十分钟后，班长去借书，你到图书馆门外休息。这时，你的同学来了。她在图书馆门口见到你，你们俩开始聊天。
聊天期间，你想告诉她班长已经到了这件事，你会怎么说？
When you arrive at the library, the class monitor is already there. Ten minutes later, your classmate arrives just when the class monitor goes to check out some books and you take a break outside the library. She meets you and chats with you.
In the course of the conversation, you’d like to tell her that the class monitor has arrived. What would you say?

包裹
Package context

五天前，你和你的室友在网上买了一个东西。快递员打电话说包裹会星期五送到你们家。今天是星期五，但是你的室友要去上班，所以你在家里等快递员。
Five days ago, you and your housemate bought something on the internet. The deliveryman told you he would deliver the package to your house on Friday. Today is Friday and your housemate has to go to work, so you wait for the deliveryman at home.

1. 当你的室友晚上回来时，她说："我猜快递员已经来过了。"
   你对快递员来过的事实予以肯定，你会怎么说？
   When your housemate comes back at night, she comments:
   Wǒ cāi kuàidìyuán yǐjīng lái guò le.
   I guess delivery man already come PART PART
   'I guess the delivery man has come.'
   In order to confirm that the delivery man has already come, what would you say?

2. 当你的室友傍晚回来时，快递员还没有送包裹来。她进门时看到了你，并说："我猜快递员已经来过了。"
   你对快递员来过的事实予以否定。你会怎么说？
   When your housemate comes back in the evening, the deliveryman hasn't come. When entering the apartment, she sees you and comments:
   Wǒ cāi kuàidìyuán yǐjīng lái guò le.
   I guess delivery man already come PART PART
   'I guess the delivery man has already come.'
   In order to deny that the delivery man has already come, what would you say?

3. 当你的室友下午回来时，在你们经常放包裹的客厅的茶几上没看到包裹，于是说道："看来快递员还没有来。"
   你对快递员还没来的事实予以肯定，你会怎么说？
   When your housemate comes back in the afternoon, she doesn't see the package on the tea table in the living room where you usually put packages. She sees you and says:
   Kànlái kuàidìyuán hái méiyǒu lái.
   seem delivery man still no have come
   'It seems that the deliveryman hasn't come yet.'
   In order to confirm that the deliveryman hasn't come, what would you say?

4. 当你的室友下午回来时，没在你们经常放包裹的客厅的茶几上看到包裹。但事实上快递员已经把包裹送来了，只是你把包裹拿到饭厅去了，没有拿回客厅。因为你的室友没有看到包裹，她说："看来快递员还没有来。"
   你对她的话予以反对，你会怎么说？

When your housemate comes back in the afternoon, she doesn’t see the package on the tea table in the living room where you usually put packages. In fact, the deliveryman has come, but you have taken the package to the dining room and haven’t brought it back to the living room. As your housemate doesn’t see the package, she says:

Kànlái kuàidìyuán hái méiyǒu lái.

seem delivery man still no have come

‘It seems that the deliveryman hasn’t come yet.’

In order to deny what she said, what would you say?

5. 当你的室友晚上回来时，她说：“快递员已经来过了，是吗？”
   你对快递员已经来过的事实予以肯定，你会怎么说？

   When your housemate comes back at night, she says:

   Kuàidìyuán yǐjīng lái guò le, shì ma?

   delivery man already come PART PART is QPART

   ‘The deliveryman has already come，hasn’t he?’

   In order to confirm that the deliveryman has already come, what would you say?

6. 当你的室友傍晚回来时，快递员还没有送包裹来。她进门时看到了你并说：“快递员已经来过了，是吗？”
   你对快递员来过的事实予以否定。你会怎么说？

   When your housemate comes back in the evening, the deliveryman hasn’t come. When entering the apartment, she sees you and says:

   Kuàidìyuán yǐjīng lái guò le, shì ma?

   delivery man already come PART PART is QPART

   ‘The delivery man has already come，hasn’t he?’

   In order to deny that the deliveryman has come, what would you say?

7. 当你的室友下午回来时，在你们经常放包裹的客厅的茶几上没看到包裹，所以她说：“快递员还没有来，是吗？”
   你对快递员还没来的事实予以肯定，你会怎么说？

   When your housemate comes back in the afternoon, she doesn’t see the package on the tea table in living room where you usually put packages. So she says:

   Kuàidìyuán hái méiyǒu lái, shì ma?

   delivery man still no have come is QPART

   ‘The deliveryman hasn’t come yet, has he?’

   In order to confirm that the deliveryman hasn’t come, what would you say?

8. 当你的室友下午回来时，没在你们经常放包裹的客厅的茶几上看到包裹。但事实上快递员已经把包裹送来了，只是你把包裹放到饭厅去了，没有拿回客厅。因为你的室友没有看到包裹，她说：“快递员还没有来，是吗？”
   你对她的话予以反对，你会怎么说？

   When your housemate comes back in the afternoon, he doesn’t see the package on the tea table in the living room where you usually put packages. In fact, the

deliveryman has come, but you have taken the package to the dining room and haven't brought it back to the living room. As your housemate doesn't see the package, she says:

Kuàidìyuán hái méiyǒu lái, shì ma?
delivery man still no have come is QPART
'The deliveryman hasn't come yet, has he?'
In order to deny what she said, what would you say?

9. 快递员下午已经把包裹送来了，现在包裹已经在家里了。当你的室友回家时，你们俩开始聊天。
聊天期间，你想告诉你的室友快递员已经来过了这个事情，你会怎么说？
The deliveryman has delivered the package. Now the package is in your house. When your housemate comes back and enters the apartment, she chats with you.
In the course of the conversation, you'd like to tell her that the deliveryman has come. What would you say?

婚礼
Wedding context

今天是你同事结婚的日子，你和你的好朋友都要去参加，你们会送红包给新人。你的好朋友因她的私事会迟到一会儿，她让你帮她把她的红包先送给新人。你已经到了举行婚礼的酒店，你的好朋友办完事情会赶来酒店，但是会迟到一会儿。
Today is your colleague's wedding day. You and your friend are going to attend the wedding and give the newlyweds the red envelopes. Your friend will be a little late for her private affairs. She asked you to help her to give her red envelope to the newlyweds. You have arrived at the hotel where the wedding will be held, your friend will be a little late after finishing her private affairs.

1. 当你的好朋友到举行婚礼的地点时，你已经把红包给新人了。你的好朋友见到你并说：“我猜你已经把红包给新人了。”
你对你给了红包的事实予以肯定，你会怎么说？
When your friend arrives at the place where the wedding will be held, you have given the red envelopes to the newlyweds. Your friend sees you and says:

Wǒ cāi nǐ yǐjīng bǎ hóngbāo gěi xīnrén le.
I guess you already prep. red envelope give newlywed PART
'I guess you have given the red envelopes to the newlyweds.'
In order to confirm that you have given them to the newlyweds, what would you say?

2. 当你的好朋友到举行婚礼的地点时，你还没把红包给新人。你的好朋友见到你并说：“我猜你已经把红包给新人了。”
你对你给了红包的事实予以否定，你会怎么说？
When your friend arrives at the place where the wedding will be held, you haven't

given the red envelopes to the newlyweds. Your friend sees you and says:

Wǒ cāi nǐ yǐjīng bǎ hóngbāo gěi xīnrén le.

I guess you already prep. red envelope give newlywed PART

'I guess you have given the red envelopes to the newlyweds.'

In order to deny that you have given the red envelopes to the newlyweds, what would you say?

3. 当你的好朋友到举行婚礼的地点时，你还没把红包给新人。当她到的时候，你手里正拿着红包，她说："看来你还没把红包给新人。"
你对你还没把红包给新人的事实予以肯定，你会怎么说？
When your friend arrives at the place where the wedding will be held, you haven't given the red envelopes to the newlyweds. Seeing the red envelopes in your hand, she says:
Kànlái nǐ hái méi bǎ hóngbāo gěi xīnrén.
seem you still no prep. red envelope give newlywed
'It seems that you haven't given the red envelopes to the newlyweds.'
In order to confirm that you haven't given them to the newlyweds, what would you say?

4. 当你的好朋友到举行婚礼的地点时，你已经把红包给新人了。但你的手里拿着你另外两个朋友的红包，因为他们去上卫生间了，让你帮忙拿着。当你的好朋友到的时候，看到你手里的红包并说："看来你还没把红包给新人。"
你对她的话予以反对，你会怎么说？
When your friend arrives at the place where the wedding will be held, you have given the red envelopes to the newlyweds. But there are two red envelopes in your hand which two of your friends asked you to hold while they went to the bathroom. Seeing the red envelopes in your hand, she says:
Kànlái nǐ hái méi bǎ hóngbāo gěi xīnrén.
seem you still no prep. red envelope give newlywed
'It seems that you haven't given the red envelopes to the newlyweds.'
In order to deny what she said, what would you say?

5. 当你的好朋友到举行婚礼的地点时，你已经把红包给新人了。她见到你并问："红包已经给新人了，是吗？"
你对你把红包给新人的事实予以肯定，你会怎么说？
When your friend arrives at the place where the wedding will be held, you have given the red envelopes to the newlyweds. Your friend sees you and asks:
Hóngbāo yǐjīng gěi xīnrén le, shì ma?
red envelope already give newlywed PART is QPART
'You have given the red envelopes to the newlyweds, haven't you?'
In order to confirm that you have given the red envelopes to the newlyweds, what would you say?

6. 当你的好朋友到举行婚礼的地点时，你还没把红包给新人。她见到你并问：“红包已经给新人了，是吗？”
你对你把红包给新人的事实予以否定，你会怎么说？
When your friend arrives at the place where the wedding will be held, you haven't given the red envelopes to the newlyweds. Your friend sees you and asks:
Hóngbāo yǐjīng gěi xīnrén le, shì ma?
red envelope already give newlywed PART is QPART
'You have given the red envelopes to the newlyweds, haven't you?'
In order to deny that you have given them to the newlyweds, what would you say?

7. 当你的好朋友到举行婚礼的地点时，你还没把红包给新人。当她到的时候，你手里正拿着红包，她问：“你还没把红包给新人，是吗？”
你对你还没把红包给新人的事实予以肯定，你会怎么说？
When your friend arrives at the place where the wedding will be held, you still haven't given the red envelopes to the newlyweds. Seeing the red envelopes in your hand, she asks:
Nǐ hái méi bǎ hóngbāo gěi xīnrén, shì ma?
you still no prep. red envelope give newlywed is QPART
'You haven't given the red envelopes to the newlyweds, have you?'
In order to confirm that you haven't given them to the newlyweds, what would you say?

8. 当你的好朋友到举行婚礼的地点时，你已经把红包给新人了。但你的手里拿着你另外两个朋友的红包，因为他们去上厕所了，让你帮忙拿着。当你的好朋友到的时候，看到你手里的红包并问：“你还没把红包给新人，是吗？”
你对她的话予以反对，你会怎么说？
When your friend arrives at the place where the wedding will be held, you have given the red envelopes to the newlyweds. But there are two red envelopes in your hand which two of your friends asked you to hold while they went to the bathroom. Seeing the red envelopes in your hand, she says:
Nǐ hái méi bǎ hóngbāo gěi xīnrén, shì ma?
you still no prep. red envelope give newlywed is QPART
'You haven't given the red envelopes to the newlyweds, have you?'
In order to deny what she said, what would you say?

9. 当你的好朋友到举行婚礼的地点时，你正在酒店门口休息，你已经把红包给新人了。她在酒店门口见到你，你们开始聊天。
聊天期间，你想告诉她你已经把红包给了新人这个事，你要怎么说？
When your friend arrives at the hotel where the wedding will be held, you are taking a rest at the door of the hotel. You have given the red envelopes to the newlyweds. She sees you and chats with you.
In the course of the conversation, you'd like to tell her that you have given the red envelopes to the newlyweds. What would you say?
